# Differentiation of Mesenchymal Stem Cells from Human Umbilical Cord Tissue into Odontoblast-Like Cells Using the Conditioned Medium of Tooth Germ Cells *In Vitro*

**DOI:** 10.1155/2013/218543

**Published:** 2013-05-13

**Authors:** Tian Xia Li, Jie Yuan, Yan Chen, Li Jie Pan, Chun Song, Liang Jia Bi, Xiao Hui Jiao

**Affiliations:** ^1^Department of Prosthodontics, The First Affiliated Hospital of Harbin Medical University, Harbin, Heilongjiang 150001, China; ^2^Department of Oral Health Sciences, The First Affiliated Hospital of Harbin Medical University, Harbin, Heilongjiang 150001, China; ^3^The Key Laboratory of Cell Transplantation of the Ministry of Health and Department of General Surgery, The First Affiliated Hospital of Harbin Medical University, Harbin, Heilongjiang 150001, China; ^4^Department of Dentistry, The Fourth Affiliated Hospital of Harbin Medical University, Harbin, Heilongjiang 150001, China; ^5^Department of Oral Maxillofacial Surgery, The First Affiliated Hospital of Harbin Medical University, Harbin, Heilongjiang 150001, China

## Abstract

The easily accessible mesenchymal stem cells in the Wharton's jelly of human umbilical cord tissue (hUCMSCs) have excellent proliferation and differentiation potential, but it remains unclear whether hUCMSCs can differentiate into odontoblasts. In this study, mesenchymal stem cells were isolated from the Wharton's jelly of human umbilical cord tissue using the simple method of tissue blocks culture attachment. UCMSC surface marker expression was then evaluated for the isolated cells using flow cytometry. The third-passage hUCMSCs induced by conditioned medium from developing tooth germ cells (TGC-CM) displayed high alkaline phosphatase (ALP) levels (*P* < 0.001), an enhanced ability to proliferate (*P* < 0.05), and the presence of mineralized nodules. These effects were not observed in cells treated with regular medium. After induction of hUCMSCs, the results of reverse transcriptional polymerase chain reaction (PCR) indicated that the dentin sialophosphoprotein (DSPP) and dentin matrix protein 1 (DMP1) genes were significantly tested. Additionally, dentin sialoprotein (DSP) and DMP1 demonstrated significant levels of staining in an immunofluorescence analysis. In contrast, the control cells failed to display the characteristics of odontoblasts. Taken together, these results suggest that hUCMSCs can be induced to differentiate into odontoblast-like cells with TGC-CM and provide a novel strategy for tooth regeneration research.

## 1. Introduction

Tooth loss, caused by dental caries, periodontal diseases, injuries, or a variety of genetic disorders, is one of the most common human diseases. Numerous studies have addressed stem cell-based tooth tissue engineering strategies aimed at reconstituting a bioengineered tooth to treat tooth loss. With their significant capacity for self-renewal and pluripotent differentiation, mesenchymal stem cells (MSCs) are used as an important type of seed cells for tissue engineering and regenerative medicine. Compared with other tissues (adipose tissue, cord blood, synovial fluid, dental pulp, dermis, and muscle), bone marrow (BM) has been identified as a common source of MSCs for both experimental and clinical applications, and BMMSCs are also capable of differentiating into odontoblast-like cells [1–6]. However, BM collection is a highly invasive procedure and may lead to a variety of complications and cell contamination. Furthermore, the proliferative capacity and differentiation potential of BM cells decline with increasing age [7, 8]. As these problems have remained barriers to the clinical application of BMMSCs, more suitable and easily obtainable stem cells are required to further tooth regeneration research.

Human umbilical cord (UC) tissue has been suggested to represent another promising source of MSCs [9, 10]. During pregnancy, the mother and fetus are connected by the umbilical cord, which is comprised of umbilical vessels (two arteries and one vein) and a specialized mucous connective tissue called Wharton's jelly, all covered by the amniotic epithelium [11]. Thus, UC tissue, an inevitably discarded product of full-term delivery, is a relatively rich tissue source [12]. The isolation of human umbilical cords is noninvasive, painless, and harmless for both the mother and the infant and therefore avoids any ethical or technical controversy. In addition, it has been found that MSCs derived from human umbilical cord Wharton's jelly, which express certain embryonic stem cell (ESCs) markers (such as NANOG, DNMT3B, and GABRB3), are more primitive than those isolated from other tissue sources [13]. As compared to BMMSCs, UCMSCs are believed to manifest a greater proliferative potential and capacity to differentiate into various cell types, such as chondrocytes, adipocytes, osteoblasts, cardiomyocytes, dermal fibroblasts, neurons, and endothelial cells, depending on the inductive media [13–18]. The “stem cell niche,” which is considered to be the native microenvironment of stem cells, is thought to maintain the characteristics and functions of stem cells, and to guide differentiation [19]. Previous studies have confirmed that TGC-CM contains a series of complex soluble signaling molecules and growth factors secreted by the epithelial and mesenchymal cells of the tooth germ cells and can create a potent odontogenic microenvironment [20, 21]. Furthermore, there is accumulating evidence that TGC-CM can also meet many needs for the differentiation of odontogenic cells such as dental pulp stem cells (DPSCs) and stem cells from human exfoliated deciduous [21–24]. TGC-CM has also been shown to promote odontogenic lineage development in nonodontogenic cells, such as dermal multipotent cells, adipose-derived stem cells and follicle dermal papilla mesenchymal cells [12, 25, 26]. These results, together with the advantages of hUCMSCs, prompted us to investigate whether hUCMSCs could be induced to differentiate along the odontoblast lineage when exposed to TGC-CM.

The main goals of this study were to ascertain whether the MSC from UC Wharton's jelly had the capacity to synthesize the specific markers of functional odontoblast when cultured in TGC-CM *in vitro*. Overall, our data demonstrate for the first time that UC-derived MSCs could potentially differentiate into odontoblast-like cells in an odontogenic microenvironment *in vitro*; we explore an optimal alternative tissue source of MSCs for dental regeneration engineering.

## 2. Materials and Methods

### 2.1. Isolation and Culture of hUCMSCs

With parental consent, human umbilical cords were collected from full-term newborn infants delivered by cesarean section at the Department of Gynecology and Obstetrics at The First Affiliated Hospital of Harbin Medical University. All of the procedures were approved by the institutional ethics committee of the university. Human UCMSCs were isolated using the tissue block culture attachment method, as previously reported [27, 28], and cultured in a 37°C incubator with 5% CO_2_ in Dulbecco's modified Eagle medium/F-12 (DMEM/F-12, HyClone, Logan, UT, USA) containing 15% fetal bovine serum (FBS, Gibco, Grand Island, NY, USA), 100 units/mL penicillin, and 100 mg/mL streptomycin (Beyotime, Shanghai, China). Five days later, the tissue blocks were removed, and the medium was replaced. The medium was replaced every 3 days thereafter. When the cells reached 80% confluence, they were digested with 0.25% trypsin/1 mM ethylenediaminetetraacetic acid (EDTA) (HyClone, Logan, UT, USA) and seeded into 25 cm^2^ culture flasks (Costar, Cambridge, MA, USA) at 5 × 10^3^ cells/cm^2^ for propagation and culture.

### 2.2. Investigation of Cell Surface Markers Expression by Flow Cytometry

To determine the phenotype of the hUCMSCs, primary cells were collected and washed twice in phosphate-buffered saline (PBS; Gibco, Grand Island, NY, USA). Thereafter, the cells were digested and resuspended at a concentration of 1.0 × 10^6^ cells/mL. For staining, 20 *μ*L of each of the following human antibodies was added to 100 *μ*L of the cell suspension: CD45-PE monoclonal antibody (mAb); CD73-PE mAb; CD105-FITC mAb; CD90 (Thy1)-FITC mAb; and CD34-APC mAb (Cat: number 304007; Cat: number 344003; Cat: number 323203; Cat: number 328107; Cat: number 343509, Biolegend, California, USA). Negative control staining was performed using the corresponding isotype control antibodies (FITC, Mouse IgG1, Cat: number 400109; PE, Mouse IgG1, Cat: number 400111; APC, Mouse IgG1, and Cat: number 400119; Biolegend). The samples were incubated at 37°C for 30 min, centrifuged, washed twice with PBS, and examined by Elite ESP flow cytometry (Beckman Coulter Inc., Fullerton, CA, USA).

### 2.3. Determination of Cell Differentiation Capacity

Third-passage hUCMSCs were digested, collected, and plated at a density of 3 × 10^3^ cells/cm^2^ and subjected to adipogenic, neuron-like cell and osteogenic differentiation *in vitro*. Normal UC-derived cells were used as negative controls in the differentiation studies.

To verify the adipogenic differentiation potential, the cultured cells were induced in culture medium supplemented with 0.5 *μ*M isobutyl-methylxanthine (IBMX, Sigma-Aldrich, PA, USA), 50 *μ*M indomethacin (Sigma-Aldrich), 0.5 *μ*M dexamethasone (Sigma-Aldrich), and 5 *μ*g/mL insulin (Sigma-Aldrich). Adipogenic differentiation was determined by detecting the intracellular accumulation of lipid granules with Oil Red O staining in the third week of culture. 

The neuron-like cell differentiation potential was investigated in the presence of neural inductive medium containing 2% dimethylsulfoxide (DMSO), 200 *μ*M butylated hydroxyanisole (BHA), 10 *μ*M forskolin, 0.1 mM/L *β*-mercaptoethanol (*β*-ME), and 1 *μ*M hydrocortisone. After 10 days, the expression of nestin (Chemicon, Temecula, CA, USA) was confirmed by immunofluorescent staining.

For the induction of osteogenic differentiation, UC cells were treated with osteogenesis-inductive medium supplemented with 50 *μ*g/mL ascorbate-2 phosphate, 10 mM *β*-glycerophosphate (Sigma-Aldrich), and 0.1 *μ*M dexamethasone and cultured for 21 days. Osteogenesis was assessed using Alizarin Red S staining as an indicator of mineralized calcium phosphate accumulation.

### 2.4. Preparation of Tooth Germ Cell-Conditioned Medium

All experimental procedures were performed according to the Guidelines of the Animal Care Committee of Harbin Medical University. The TGC conditioned medium was prepared as described previously [26]. Briefly, the tooth germs were isolated from the lower molars of 1-day postnatal Sprague-Dawley (SD; HMU Medical Laboratory Animal Center, Harbin, China) rats and minced into less than 1 mm^3^ pieces in PBS. The fragments were digested with 3 mg/mL type I collagenase (GIBCO, Grand Island, NY, USA) for 40–60 min at 37°C. The TGCs were collected by centrifugation and washed twice in DMEM/F-12 containing 10% FBS. Single-cell suspensions were obtained by filtration through a 70 *μ*m strainer and were then washed with DMEM/F-12 supplemented with 10% FBS, 2.5 mM glutamine, 100 units/mL penicillin G and 100 mg/mL streptomycin. The cells were then placed into 75 cm^2^ culture flasks at a concentration of 1 × 10^5^ cells/mL and grown in 5% carbon dioxide at 37°C. The culture medium for the primary TGCs containing both epithelial and mesenchymal cells was changed every day when confluence was 70%. Once contact inhibition occurred, the supernatant was collected and filtered using a 0.22 *μ*m Millipore strainer (Carrigtwohill Co., Cork, Ireland). The supernatant was mixed with an equal volume of fresh DMEM/F-12 supplemented with 10% FBS and used as TGC-conditioned medium (TGC-CM) for the UCMSC cultures. DMEM/F-12 supplemented with 10% FBS was used as control medium. 

### 2.5. Cell Cycle Analysis

After 7 days of culture in the presence of TGC-CM, the hUCMSCs were collected by digestion with trypsin/EDTA and were washed twice with PBS. The cells were then resuspended as a single cell suspension in 1 mL PBS and fixed with 2 mL cold 70% dehydrated alcohol at 4°C for 24–48 h. After washing again, the cell suspensions were stained with propidium iodide (100 mg/mL; Sigma-Aldrich) at 4°C for 30 min and subjected to Elite ESP flow cytometry for cell cycle analysis. One million cells were evaluated per sample. Human UCMSCs cultured in normal medium were used as controls.

### 2.6. ALP Activity Assay

UC-derived cells at passage 3 were trypsinized and suspended in TGC-CM at a density of 1 × 10^3^ cells/well in 96-well plates. The alkaline phosphatase activity of the TGC-CM-treated hUCMSCs was detected on days 3, 6, 9, 12, 15, 18, and 21 using an ALP assay kit (Jiancheng Co., Nanjing, China) according to the manufacturer's instructions. The cells were washed in PBS and lysed with 0.05% Triton X-100 solution. The absorbance of each well was measured spectrophotometrically at 520 nm with a microplate reader. Human UCMSCs cultured in regular DMEM/F-12 media were used as a control.

### 2.7. Alizarin Red S Staining for Mineralized Nodule Formation

Third-passage cells were seeded into 6-well plates (Costar, Cambridge, MA, USA) at an initial density of 5 × 10^4^ cells/well and cultured in either TGC-CM or regular DMEM/F-12. The cells were rinsed twice with PBS and fixed in 95% ethanol for 30 min at room temperature. The fixative was carefully removed, and the cells were gently rinsed three times with distilled water, followed by staining with 0.1% Alizarin Red S (Sigma-Aldrich) at 37°C for 30 min.

### 2.8. Immunofluorescent Staining

After 14 days of exposure to TGC-CM, the third-passage hUCMSCs were seeded into 24-well culture plates at a density of 2 × 10^5^ cells/well and fixed with 4% polyoxymethylene for 20 min at room temperature. Immunocytochemical analysis was performed according to the recommended protocol from the manufacturer. The applied antibodies included rabbit anti-DSP (1 : 100; Santa Cruz Biotechnology, CA, USA) and goat anti-DMP1 (1 : 50; Santa Cruz Biotechnology). The cells were incubated with rabbit anti-goat secondary antibodies conjugated to FITC (1 : 200; Santa Cruz Biotechnology) or goat anti-rabbit secondary antibodies conjugated to Rhodamine (1 : 200; Santa Cruz Biotechnology), and the cell nuclei were stained with DAPI (Beyotime Institute of Biotechnology, Jiangsu, China). The untreated hUCMSCs were used as negative controls. All antibodies were diluted in PBS.

### 2.9. Reverse Transcriptase Polymerase Chain Reaction Analysis (PCR)

Third-passage hUCMSCs at approximately 90% confluence were harvested after 14 days of exposure to TGC-CM. Total RNA was isolated according to the manufacturer's recommendations. The first-strand complementary DNA (cDNA) was synthesized using an RNA PCR kit (Takara Bio, Shiga, Japan). The polymerase chain reaction (PCR) reactions were performed as described previously [20]. The primer sequences for DSPP (dentin sialophosphoprotein), DMP1 (dentin matrix protein 1), and *β*-actin, as an internal control, were as follows: (1) DSPP-sense, 5′-CTCAGTTAGTGCCGCTGGAGA-3′ and DSPP-antisense, 5′-GAATCGTCGTTAGTGGCGTTG-3′; (2) DMP 1-sense, 5′-CTGGTATCAGGTCGGAAGAATC-3′ and DMP 1-antisense, 5′-CTCTCATTAGACTCGCTGTCAC-3′; and (3) *β*-actin sense, 5′-AGAGGGAAATCGTGCGTGAC-3′ and *β*-actin antisense, 5′-AGAGGTTTACGGATGTCAACG-3′. The following PCR cycles were used: denaturation at 94°C for 30 s, annealing at 57°C for 30 s and extension at 72°C for 30 s for 30 cycles, followed by a final incubation at 74°C for 5 min. The PCR products were analyzed by using 2% agarose gel electrophoresis and visualized by ethidium bromide staining. The expected product sizes from these primers were 472 base pairs (DSPP), 705 base pairs (DMP1), and 283 base pairs (*β*-actin).

### 2.10. Statistical Analysis

All data are expressed as the mean ± standard error of the mean (SEM). The statistical analyses were performed using a one-way analysis of variance (ANOVA) followed by Bonferroni multiple comparisons (SPSS 13.0 Software; SPSS Inc, Chicago, IL, USA). *P* values <0.05 were considered statistically significant.

## 3. Results

### 3.1. Isolation and Morphological Features of Human UC-Derived Cells

**Figure 1:**
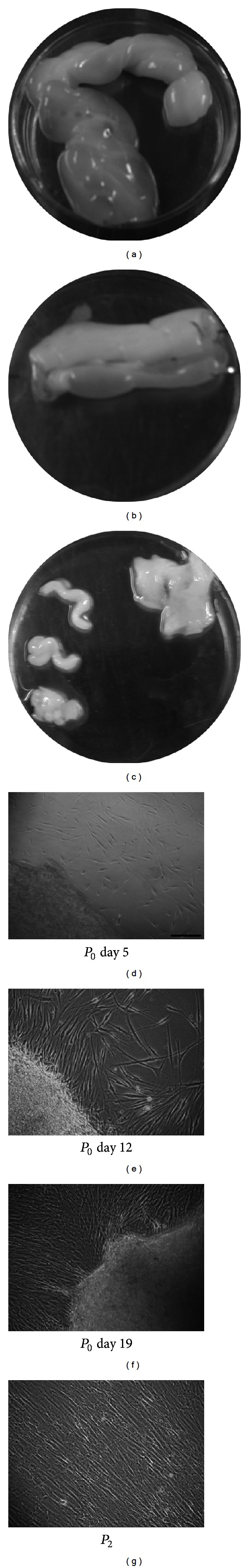
Isolative process and morphological appearance of mesenchymal stem cells (MSCs) isolated from human umbilical cords (hUCMSCs). (a) The umbilical cord (UC) was cut into pieces of approximately 4 to 5 cm in length. (b) Each segment of the UC was dissected longitudinally to expose the Wharton's jelly (WJ). (c) WJ was obtained by removing the cord vessels and the amniotic epithelium. (d) The appearance of primary hUCMSCs cultured for 5 days. (e) The appearance of primary hUCMSCs cultured for 12 days. (f) After 19 days of culture, the primary hUCMSCs reached 80–90% confluency. (g) The appearance of hUCMSCs after passaging. Scale bar = 100 *μ*m.

Using the tissue block culture attachment method, primary hUCMSCs were successfully isolated from the Wharton's jelly of umbilical cords, as depicted in Figures 1(a), 1(b), and 1(c). After 5 days in culture, it was clearly observed that the cells isolated from the Wharton's jelly of UC tissue blocks were scarce and scattered, principally exhibiting a fibroblast-like morphology (Figure 1(d)). At this time, the tissue masses that did not attach to the culture bottle were removed, and the medium was replaced. The cells gradually took on a typical fibroblastic shape after a 12-day culture period (Figure 1(e)) and tended to arrange themselves in parallel lines after 19 days (Figure 1(f)), at which time they had reached 80–90% confluency for passaging. Once passaged, the cells expanded very rapidly with no evident morphologic changes (Figure 1(g)) and were passaged approximately once every week.

### 3.2. Identification of Human UC-Derived Cells

**Figure 2:**
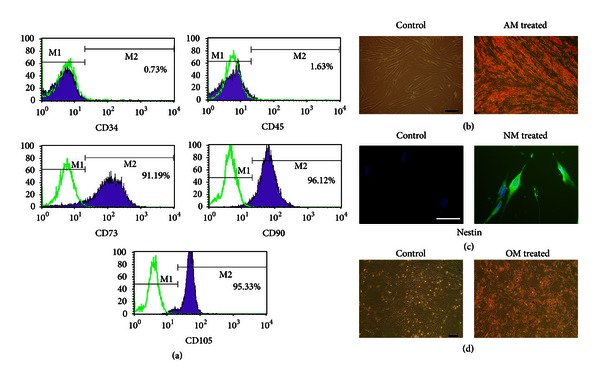
Identification of the adherent cells derived from UCs. (a) Third-passage adherent cells were positive for CD73 (91.19%), CD90 (96.12%), and CD105 (95.33%) and negative for CD34 (0.73%) and CD45 (1.63%), as analyzed by flow cytometry. (b) Human UCMSCs treated with adipocytic inductive medium (AM), but not the controls, were stained with Oil Red O. Scale bar = 100 *μ*m. (c) Immunocytochemistry revealed that stained cells were not observed in the control conditions. Human UCMSCs induced with neural-inductive medium (NM) were positive for nestin. Scale bar = 100 *μ*m. (d) Human UCMSCs in control conditions were not stained. The cells cultured in osteogenic-inductive medium (OM) were stained with Alizarin Red S. Scale bar = 100 *μ*m.

After 3 passages, the isolated cells were prepared for examination and analysis by flow cytometry. The cultured cells from the Wharton's jelly expressed high levels of the MSC surface markers CD73 and CD90 and high levels of CD105, a matrix receptor. However, the cells were negative for hematopoietic lineage markers (CD34, CD45). These results indicated that the isolated cells in this study represented hUCMSCs and were not mixed with cells of hematopoietic origin (Figure 2(a)). Consistent findings concerning the immunophenotypic characteristics of hUCMSCs have been reported by other investigators [28, 29].

To verify the multipotency of the hUCMSCs, the cells were assessed for their adipogenic, neural, and osteogenic differentiation capacities *in vitro*. When the cells were cultured in adipogenic medium for 21 days, a number of lipid vacuoles, which are considered a unique characteristic of the adipogenic phenotype, were observed following staining with Oil Red O solution (Figure 2(b)). After 10 days of neuronal induction, differentiated cells exhibiting neural-like morphology and cells positive for nestin expression, which is generally used as a specific marker for early neural progenitors, could be observed in the hUCMSC cultures (Figure 2(c)). As depicted in Figure 2(d), the deposition of calcium in the cells, which is universally used to identify osteogenesis, was noticeable and could be visualized by Alizarin Red S staining after 21 days of induction, while the cells cultured in the control media were not stained by Alizarin red S (Figure 2(d)).

### 3.3. Cell Cycle Assay of TGC-CM-Induced hUCMSCs

**Figure 3:**
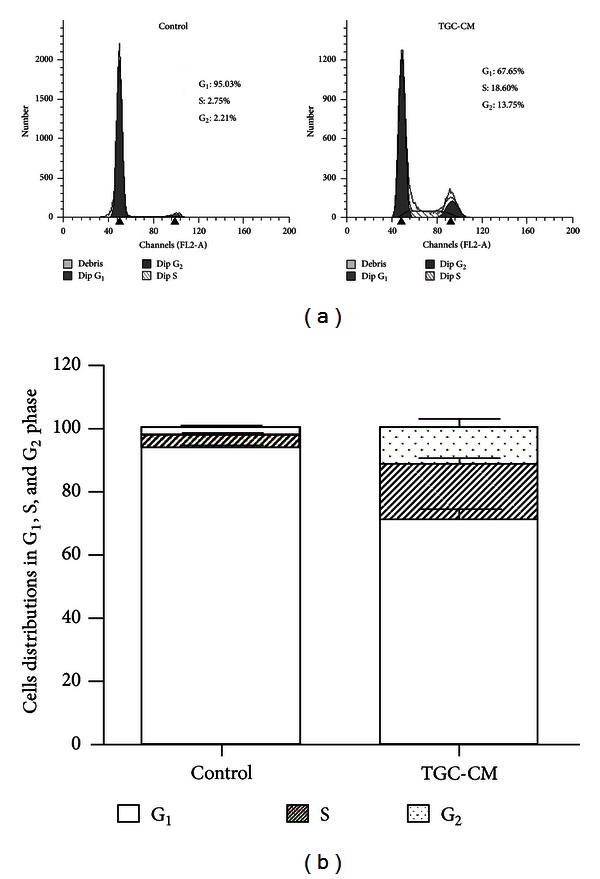
Effects of TGC-CM on the cell cycle of hUCMSCs. (a) Representative cell cycle distributions of untreated hUCMSCs and hUCMSCs treated with TGC-CM are presented. (b) The column diagram shows the cell cycle distributions of untreated cells and cells treated with TGC-CM from three replicate experiments. *P* < 0.05.

To determine the effects of TGC-CM on the proliferative activity of hUCMSCs, cells were exposed to TGC-CM or control medium for cell cycle analysis. As shown in Figure 3, the hUCMSCs cultured in TGC-CM showed the typical distribution of three phases, with double peaks for the G_1_ and G_2_ phases, whereas the control hUCMSCs manifested only a single peak for the G_1_ phase. A significantly greater percentage of cells in the S (18.60%) and G_2_ phases (13.75%) and a lower percentage of cells in the G_1_ phase (67.65%) were observed in treated cells compared to untreated cells (S = 2.75%, G_2_ = 2.21%, G_1_ = 95.03%), suggesting that TGC-CM contributes to the enhanced proliferative ability of hUCMSCs (Figures 3(a) and 3(b)).

### 3.4. ALP Activity and Mineralization Assay of TGC-CM-Induced hUCMSCs

**Figure 4:**
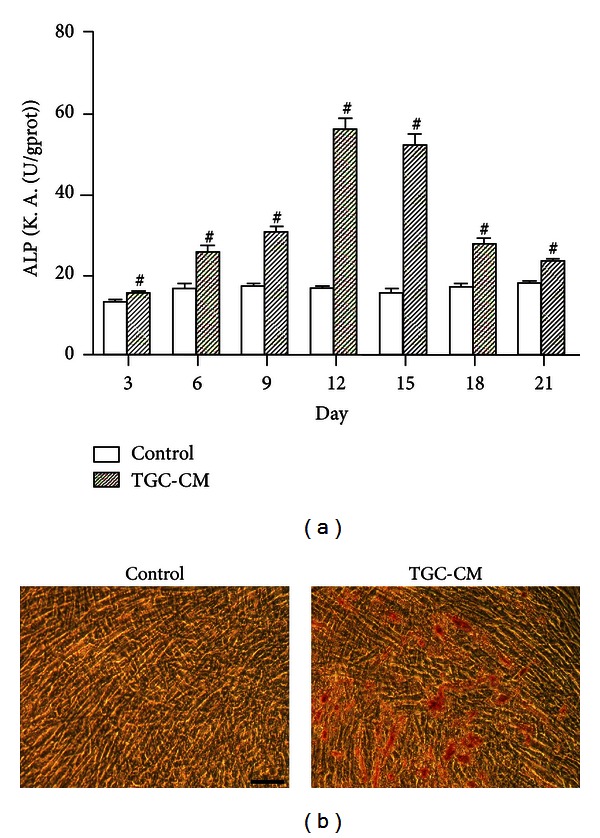
The ALP activity and mineralization potential of hUCMSCs treated with TGC-CM. (a) The column diagram presents the ALP activity of untreated cells and cells treated with TGC-CM. Each experiment was performed in triplicate. The error bars represent the mean ± SD. ^#^ *P* < 0.001. (b) Human UCMSCs cultured in control medium were not positively stained with Alizarin Red S. The cells cultured in TGC-CM for 28 days formed calcium deposits, which were stained with Alizarin Red S. Scale bar = 100 *μ*m.

To validate the odontoblast differentiation of hUCMSCs, the cells were seeded at the same density in TGC-CM or regular medium for ALP activity detection, as ALP is considered a marker of odontoblastic/osteogenic differentiation [21]. The data indicated that the ALP activity of cells treated with TGC-CM was significantly higher than that of cells treated with regular medium at each time point (*P* < 0.001). From the sixth day on, the ALP activity in the induced group significantly increased, reaching its peak level on the 12th day, but then declined gradually (Figure 4(a)). In contrast, the ALP activity in the control hUCMSCs fluctuated only slightly throughout the culture period (Figure 4(a)).

To identify the effects of TGC-CM on the mineralization ability of hUCMSCs, these cells were cultured with and without TGC-CM treatment for 4 weeks, and the deposition of calcium and phosphate was detected by Alizarin Red S staining. Microscopically, the TGC-CM-treated hUCMSCs were observed to form mineralized nodules during the 28-day culture (Figure 4(b)), while mineralization was not observed in the nontreated cells in the control group at the end of the 28-day culture, as indicated by negative Alizarin Red S staining (Figure 4(b)). The results of these experiments indicate that TGC-CM plays a crucial role in stimulating the hUCMSC mineralization potential.

### 3.5. Odontogenic Differentiation of TGC-CM-Induced hUCMSCs

**Figure 5:**
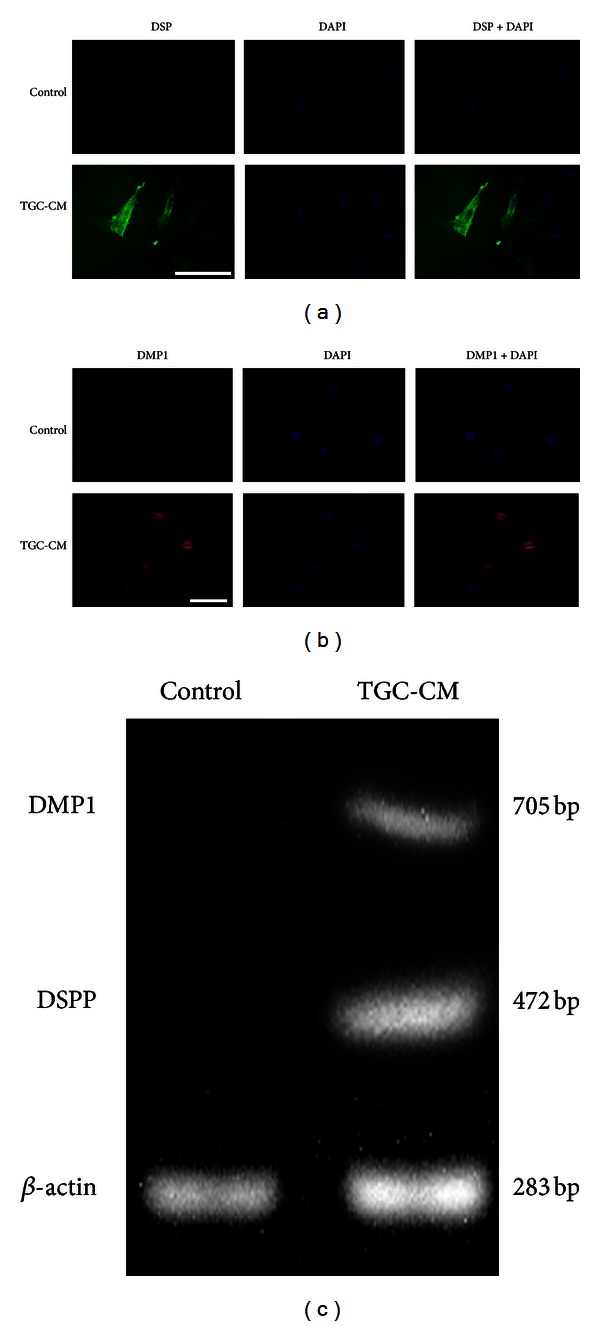
Detecting results of odontogenic differentiation of hUCMSCs induced in TGC-CM. (a) Human UCMSCs induced by TGC-CM were positively stained for DSP, but the control cells were not. Scale bar = 100 *μ*m. (b) Human UCMSCs cultured in TGC-CM were positively stained for DMP1, but the control cells were not. Scale bar = 100 *μ*m. (c) The reverse transcription PCR products indicated the expression of DSPP and DMP1 mRNA in the TGC-CM-treated UCMSCs. *β*-actin was used as an internal control for each group.

To detect characteristics of the odontoblast phenotype in induced hUCMSCs, we performed immunocytochemical staining for dentin sialoprotein (DSP) and dentin matrix protein 1 (DMP1), both of which were chosen as putative odontoblast-specific markers [30–32]. The results indicated that the hUCMSCs treated with TGC-CM for 14 days were positively stained for DSP and DMP1, while no staining was observed in the control cells using the immunocytochemical assay (Figures 5(a) and 5(b), resp.). In addition, we observed the positive staining of DMP1 in the nucleus and cytoplasm of the induced hUCMSCs, but not in the nucleolus (Figure 5(b)). These results indicated that the cells induced by TGC-CM could synthesize the DSP and DMP1 proteins.

Further evidence for expression of the odontoblast phenotype in induced hUCMSCs was provided by the evaluation of DSPP and DMP1 gene expression using reverse transcription PCR. This analysis was conducted using TGC-CM-treated and untreated cells at day 14 of culture. As shown in Figure 5(c), the expression of DSPP and DMP1 mRNA could be clearly observed in the induced hUCMSCs at day 14, while expression was not detected in the control cells. These results indicate that TGC-CM contributed to the enhanced transcription of the DSPP and DMP1 genes.

## 4. Discussion

Mesenchymal stem cells (MSCs) are capable of self-renewal and possess a high proliferative capacity and the potential for multilineage differentiation. Previous studies have reported that human umbilical cord tissue demonstrates the advantages of providing an abundant supply of cells from donors via a noninvasive procedure with a low risk of infection and can therefore be considered an attractive alternative source of MSCs [14, 15]. In the present study, cells were successfully isolated from the Wharton's jelly of human UC tissue using the tissue block culture attachment method and mechanical removal of the vessels and epithelia, as previously reported [14]. This approach for cell isolation without enzymatic digestion is simple, fast, and reproducible and maintains the cell adhesion capacity, cellular viability, and cellular function. Furthermore, this approach avoided contamination with endothelial and epithelial cells [27, 33]. The results of this study found that many of the adherent cells isolated from the Wharton's jelly of human UC displayed a typical fibroblast-like morphology. This result was consistent with those of other investigations, which reported the morphology of isolated MSCs from human UC-derived Wharton's jelly with or without enzymatic digestion [9, 27, 29]. Here, flow cytometry analysis showed that UC-derived cells expressed surface markers characteristic of mesenchymal cells without expressing hematopoietic stem cell markers, consistent with previous studies [13, 16]. Furthermore, our results indicated that the isolated cells derived from the UC retained the ability to differentiate into osteoblasts, adipocytes, and neural-like cells when incubated in the appropriate conditions. One recent report demonstrated that morphological characteristics, surface marker expression by flow cytometry, and cellular plasticity can be used to reliably identify MSCs [14]. Taking into account these considerations, the current study suggests that the adherent cells isolated from the human UC-derived Wharton's jelly are genuine hUCMSCs. 

Another recent study found that every stem cell requires an appropriate biomimicking microenvironment, which can help maintain cellular characteristics and functions and can drive stem cells towards adopting a specific cell lineage *in vitro* [12, 34]. In addition, recent reports have shown that tooth development follows a strict pattern and process, both spatially and temporally, which is modulated by continuous and intricate interactions between mesenchymal and epithelial cells [26]. The current knowledge suggests that over 300 types of genes, 100 varieties of growth and differentiation factors, and multiple relevant signaling molecules are involved in the dynamic process of tooth development. During this process, the spectrum and concentration of a large number of substances change continuously until the completion of tooth eruption is achieved [26, 35]. In addition, tooth germ cell-conditioned medium may be one of the best choices for this culture condition, as previous studies have suggested that the mixture of soluble factors secreted from the epithelial and mesenchymal cells of developing tooth germs creates an odontogenic microenvironment that could guide the differentiation of odontogenic and nonodontogenic cells along the odontoblast lineage [20, 26, 36]. Thus, through these studies, together with the pluripotency of hUCMSCs, we speculated that TGC-CM, utilized as an inductive conditioned medium, could induce hUCMSCs to differentiate into odontoblast-like cells. 

The cell cycle results from the current study demonstrated that many of the hUCMSCs treated with TGC-CM shifted from the G_1_ phase to the G_2_ or S phases, indicating that competent hUCMSCs progressively emerge as a result of sequential withdrawal from the cell cycle. According to previous studies, stem cell proliferation, involving several rounds of DNA replication can facilitate more efficient cell type conversions [19, 37]. Thus, this result also suggests that TGC-CM can promote the proliferation and differentiation of hUCMSCs. Moreover, the findings from this study displayed that TGC-CM-induced hUCMSCs had high ALP activity and formed calcified nodules, indicating that hUCMSCs may differentiate towards the odontogenic/osteogenic lineage by virtue of the TGC-CM-created microenvironment [20, 21].

Furthermore, the immunostaining results for DSP and DMP1 and the expression of the DSPP and DMP1 genes were used to assess the crucial odontogenic phenotype. The presence of DSPP has previously been found in bone, but the levels of DSPP in dentine are 400 times higher than those detected in bone [38, 39]. In addition, evidence from a previous report suggested that DSPP is synthesized by differentiated odontoblasts and that DSPP can be considered a specific marker of odontogenic differentiation [40]. DSP is one of the primary noncollagenous elements of dentine and is used as a hallmark for odontoblast differentiation and maturation. It is also widely used in the verification of odontoblasts [36, 40–42]. Furthermore, it has been suggested that DMP1 that is traditionally characterized as another specific marker for dentine and located in the cytoplasm of odontoblasts plays a crucial role in modulating both early odontoblast differentiation and late mineralization [26, 43]. However, in the current study, immunofluorescent assays showed that the majority of DMP1 expression was located in the nucleus and the minority of that was in the cytoplasm. A recent study indicates that DMP1, identified as a transcription factor in the nucleus, may participate in regulating cellular differentiation and the progression of the cell cycle; the roles of DMP1 may be related to its subcellular locations [44]. Thus, it is speculated that the role of DMP1 in the nucleus could be independent of its role in the cytoplasm. The further study is needed to elucidate these mechanisms. In addition, DSPP and DMP1 gene expressions could be detected at the mRNA level. These results indicate that hUCMSCs potentially synthesize the DSPP and DMP1 gene and have the ability to produce DSP and DMP1 proteins, revealing the characteristics of functional odontoblast. Thus, in agreement with our presumption, all these results imply that during *in vitro* culture, TGC sustains the odontogenic potential and can confer the capacity of hUCMSC to differentiate towards the odontogenic lineage.

The odontoblasts, as the primary structural and functional cells of dentinogenesis, play a key role in the process of tooth regeneration. Previous research has shown that both odontogenic stem cells and BMMSCs can differentiate into the odontoblast lineage [1]. However, odontogenic stem cells are not readily accessible, and BMMSCs age quickly. Owing to their easy attainability, high proliferative capacity, and the potential of odontogenic differentiation demonstrated in the present study, hUCMSCs may serve as an optimal candidate for future applications in the field of tooth regeneration engineering. Nevertheless, further studies are required to explore the underlying mechanism of how TGCs induce odontogenic differentiation of hUCMSCs.

## 5. Conclusion

In summary, our findings provide proof-of-principle evidence that hUCMSCs possess the multipotency to differentiate into odontoblast-like cells following exposure to TGC-CM in culture. Therefore, MSCs obtained from human umbilical cord tissue may serve as a rational candidate cell source for tooth regeneration research.
